# *In vivo* Genome Editing Therapeutic Approaches for Neurological Disorders: Where Are We in the Translational Pipeline?

**DOI:** 10.3389/fnins.2021.632522

**Published:** 2021-02-18

**Authors:** Pablo Lubroth, Gaia Colasante, Gabriele Lignani

**Affiliations:** ^1^Hummingbird Ventures, London, United Kingdom; ^2^Stem Cell and Neurogenesis Unit, Division of Neuroscience, Ospedale San Raffaele, Milan, Italy; ^3^Department of Clinical and Experimental Epilepsy, UCL Queen Square Institute of Neurology, University College London, London, United Kingdom

**Keywords:** genome editing, neurological disease, CRISPR (clustered regularly interspaced short palindromic repeat)/Cas9 (CRISPR associated protein 9)-mediated genome editing, biotech companies, translational pipeline

## Abstract

*In vivo* genome editing tools, such as those based on CRISPR, have been increasingly utilized in both basic and translational neuroscience research. There are currently nine *in vivo* non-CNS genome editing therapies in clinical trials, and the pre-clinical pipeline of major biotechnology companies demonstrate that this number will continue to grow. Several biotechnology companies commercializing *in vivo* genome editing and modification technologies are developing therapies for CNS disorders with accompanying large partnering deals. In this review, the authors discuss the current genome editing and modification therapy pipeline and those in development to treat CNS disorders. The authors also discuss the technical and commercial limitations to translation of these same therapies and potential avenues to overcome these hurdles.

## Introduction

### Genome Editing and Modification in the CNS

The possibility to introduce any desired modification in specific sites of the genome of cells, genome editing, is a longstanding ambition in biotechnology and molecular medicine and is now making precision medicine a real possibility for the treatment of genetic diseases.

A big step forward in the generation of new genome editing tools was the observation that the introduction of a double-strand-break (DSB) in the desired genomic site can strongly enhance the integration of a desired donor DNA sequence (Rouet et al., 1994). The discovery of zinc finger proteins (ZFP) dramatically changed the genome editing scenario as they are eukaryotic zinc ion-regulated small protein motifs able to bind DNA in a sequence-specific manner (Klug and Rhodes, 1987; Kim et al., 1996). When fused to transcriptional activator or repressors (ZFP-TFs), they modulate the expression of endogenous genes (Rebar et al., 2002). The next advance, the transcription activator-like effector (TALE) proteins from *Xanthomonas* bacteria, specifically recognize one single base instead of three bases (Boch et al., 2009; Moscou and Bogdanove, 2009), and can work as programmable nuclease, called TALEN (Li et al., 2011; Miller et al., 2011; Zhang et al., 2011). However, the cloning and protein engineering work for ZFNs and TALENs is complex. It requires two different effectors to cut each DNA strand as *Fok*I works as a dimer and only laboratories with extensive expertise in molecular biology could take advantage of those techniques, thus not broadly adopted by the scientific community.

Conversely, the latest CRISPR tools are much simpler and more flexible to use and require minimal molecular skills to exploit them successfully in multiple genome editing strategies (Anzalone et al., 2020). The main simplification is that DNA target specificity is ensured by short nucleic acid sequences (short guide RNA, sgRNA) rather than protein modules and their cloning is thus faster and cheaper. Beside the classic Cas9 which induce genomic DSBs favoring gene inactivation or gene correction, the nickase Cas9 is the basic platform for the base editor tools that make direct C to T or A to G conversion at the target site (Komor et al., 2016; Nishida et al., 2016; Gaudelli et al., 2017). In addition, nuclease defective Cas9 (dCas9) can become a scaffold to which different effectors can be attached to deliver specific protein functions to genomic sites, such as transcriptional activators (CRISPRa), inhibitors (CRISPRi), epigenetic factors and histone modifiers (Shi et al., 2004; Mali et al., 2013; Perez-Pinera et al., 2013; Qi et al., 2013; Chavez et al., 2015; Hilton et al., 2015; Konermann et al., 2015; Thakore et al., 2015; Amabile et al., 2016; Liu et al., 2016; McDonald et al., 2016; Morita et al., 2016; Vojta et al., 2016; Xu et al., 2016; Xiong et al., 2017; Matharu and Ahituv, 2020). The more recent fusion of the dCas9 to a modified reverse transcriptase makes possible to rewrite new genetic information into a specified DNA site; in this case the prime editing exploits a guide RNA (prime editing guide RNA, pegRNA) that provides specificity and encodes the edit to be introduced at the same time (Anzalone et al., 2019).

Advances in genome editing strategies encouraged researchers to exploit those tools for preclinical studies even in the CNS, that has always represented a major challenge. The main reason is that neurons are post-mitotic cells and HDR is mainly restricted to cycling cells, specifically in S and G2, when homologous recombination between sister chromatids normally occurs (Lin et al., 2014). However, homology-independent targeted integration (HITI) and other similar systems have been recently described as improved NHEJ-based homology-independent strategy for targeted transgene integration, still based on CRISPR/Cas9, but also efficient in post-mitotic cells (Suzuki et al., 2016).

### Preclinical Studies Using Genome Editing to Correct Neurological Diseases

ZFN and TALEN- based therapies have already been used in preclinical studies for several pathologies (Li et al., 2020). However, the technical limitations described above make these technologies challenging to be brought forward for treating CNS pathologies.

CRISPR-based genome editing to rescue neurological diseases has been recently tested in animal and *in vitro* human models. Several neurodevelopmental and neurodegenerative diseases have been tackled including Epilepsy, Autism Spectrum Disorder (ASD), Frontotemporal Dementia (FTD), Alzheimer’s, Huntington’s and Parkinson’s diseases (Yang et al., 2017; Kantor et al., 2018; Krishnan et al., 2020; Stepanichev, 2020; Turner et al., 2020; Vermilyea et al., 2020; Zhou et al., 2020). These approaches are based on either genome editing, silencing, or regulation, and they have been employed to overcome the limitations of classical gene therapy approaches.

Gene silencing and deletion of pathogenic repeats have been tested in animal and human models of Angelman Syndrome (AS), Fragile X syndrome (FXS), FTD and Alzheimer’s (Park et al., 2015, 2019; Xie et al., 2016; Gyorgy et al., 2018; Krishnan et al., 2020; Wolter et al., 2020). Although the results of these studies are promising showing rescue of the pathologies *in vitro* and *in vivo*, there are still preclinical tests to be performed in order to translate these approaches to the clinic. Some examples are the downstream effects of silencing a gene in a fully developed mature brain (Shitik et al., 2020), or the potential side effects of AAV integration in the DSBs (Wolter et al., 2020). Single hit mediated gene silencing of a pathogenic allele, as well as deletion of aberrant repeats, could have less impact on the immunological system. The disadvantages to these approaches are two-fold: the potential CRISPR-mediated off-target effects resulting in permanent changes to the genome and the delivery of these tools to patients. There is a massive ongoing effort to find better bioinformatic tools to predict off-target effects and in developing new delivery strategies to widely target CNS (Cota-Coronado et al., 2019).

CRISPRa, for example, has been already tested in *in vivo* animal models of neurodevelopmental and acquired epilepsies, and obesity (Matharu et al., 2019; Colasante et al., 2020a,b; Yamagata et al., 2020). These studies showed, for the first time, a long-lasting effect of endogenous gene upregulation either rescuing haploinsufficiency or modifying neuronal properties to treat pathological symptoms. Although there is great potential for effectively treating several CNS pathologies with CRISPRa, some hurdles for using this technology in humans still has to be addressed. These include the potential immunological response of the brain to long-term expression of dCAS9 (Crudele and Chamberlain, 2018) and a more efficient delivery (e.g., using smaller dCAS9).

On the other hand, the possibility of using genome editing to correct the pathological mutations is still an attractive prerogative of the CRISPR systems. Although the post-mitotic neuronal genome is difficult to modify, some recent techniques (Suzuki et al., 2016; Nishiyama et al., 2017; Yeh et al., 2019) allow gene modification in mature brain cells. In these studies, a successful insertion of new DNA in the genome of neurons has been shown to mildly rescue pathological conditions. Indeed, the main limitation is the low efficiency of the modifications that need to be addressed and improved before moving toward the clinic.

Furthermore, CRISPR base editors and CRISPR prime editing hold the potential to further improve the treatments for neurological diseases (Gaudelli et al., 2017; Anzalone et al., 2019; Duarte and Deglon, 2020). They are still behind in the preclinical pipeline due to the difficulties in the delivery of these constructs and the validation of the off-target effects. However, their ability to correct single mutations (Base editor) or longer DNA sequences (Prime) with high efficiency, without indels, is promising for future translational treatments. Recently, it has been shown that CRISPR base editing can be successfully employed *in vivo* to treat Amyotrophic Lateral Sclerosis (ALS) (Lim et al., 2020), splitting the base editors with an intein-mediated *trans-*splicing system, but the efficiency is still low.

Overall, all these different CRISPR-based technologies have been tested either in animal or *in vitro* human models, revealing an unprecedented potential for translation. The next steps are the refinement of the tools, in terms of delivery, efficiency and off-target effects in order to enable the development of an extensive commercial pipeline.

## Discussion

### The Current Therapeutic Pipeline to in Human Genome Editing

Despite the achievements in preclinical studies, therapeutic use of genome editing in the CNS is still in its infancy. Even though there are nine active clinical trials using *in vivo* genome editing^1^ (Hirakawa et al., 2020) none of them are to treat a CNS indication. Yet the potential of these technologies to treat CNS disorders is of great interest to pharmaceutical companies as seen from their pre-clinical pipelines (Table 1).

**TABLE 1:** Companies with *in vivo* genome editing and regulation assets at preclinical stage [search on November 10, 2020].

Company	Genome editing system	Approach	Affected Tissue/Organ/Therapeutic Area	Indication	Delivery	Target or Gene Delivered
Sangamo Therapeutics/Biogen	ZFP-TF	Gene downregulation	CNS	Tauopathies	AAV	Tau
Sangamo Therapeutics/Biogen	ZFP-TF	Gene downregulation	CNS	Synucleinopathies (Inc., Parkinson’s Disease)	AAV	Alpha-synuclein
Sangamo Therapeutics/Biogen	ZFP-TF	Gene downregulation	PNS and/or CNS	Neurological (Inc., a neuromuscular indication)	AAV	Unknown
Sangamo Therapeutics/Pfizer	ZFP-TF	Gene downregulation	CNS	ALS/FTD	AAV	Mutant C9ORF72
Sangamo Therapeutics/Takeda	ZFP-TF	Gene downregulation	CNS	Huntington’s Disease	AAV	Mutant HTT
Sangamo Therapeutics	ZFP-TF	Gene downregulation	CNS	Prion	AAV	Unknown
Sangamo Therapeutics/Novartis	ZFP-TF	Gene downregulation	CNS	Neurodevelopmental Disorders (Inc., Autism Spectrum Disorder)	AAV	Unknown
Editas Medicine/Asklepios Biopharmaceutical	CRISPR/Cas9	Unknown	PNS and/or CNS	Neurological	AAV	Unknown
Beam Therapeutics	CRISPR/dCas (base editor)	Correction or Silencing	CNS	Unknown	AAV	Unknown

*Public pre-clinical pipelines of biopharmaceutical companies using *in vivo* genome editing to treat CNS disorders. Queried the pipelines of genome editing companies and used search engines to find companies with publicly available information on its pipelines. Companies using genome editing and regulation technologies that do not publicize their pipelines are not shown.*

Among the several biotech companies involved in genome editing and regulation, Sangamo Therapeutics (Sangamo), Editas Medicine and Beam Therapeutics are the only ones that have publicly stated their pipelines on *in vivo* genome editing therapies for the CNS. Interestingly, Beam Therapeutics, which uses CRISPR/Cas9-based base editing, has an undisclosed CNS project.

Sangamo and Biogen are co-developing up to another ten therapeutic candidates targeting a neurological indication using ZFP-TF, with one of the assets targeting a neuromuscular indication, whereas Editas Medicine and Asklepios BioPharmaceutical (AskBio) are developing a therapy utilizing AAV-CRISPR-Cas9. AskBio was acquired by Bayer in October 2020, positioning this large pharmaceutical company in the gene therapy and genome editing space^2^. Sangamo has disclosed that its pipeline includes therapies for tauopathies, synucleinopathies, Huntington’s disease, neurodevelopmental disorders, prion disease and ALS/FTD^3^.

On the other hand, other genome editing companies such as CRISPR Therapeutics, Intellia Therapeutics and Precision Biosciences have not entered the CNS space or have not yet disclosed their candidates.

Although there is great potential of prime editing it is too early for this technology to be added to commercial pipelines. Indeed, there are currently no publicized therapy assets using prime editing. To be noted, Beam Therapeutics licensed the IP for prime editing from Prime Medicine^4^.

*Why are there still only few in vivo genome editing therapeutic programmes for the CNS?* This is due to technical and commercial limitations. Biotechnology companies seek the indications with the largest patient population that are not adequately treated by current therapies. In this equation, companies also compute the risk of failure at a technical level. Delivering *in vivo* genome editing therapies to the CNS is technically harder than to other organ systems, which increases the risk of failure. In addition, CNS indications often have a more complex etiology than oncology or monogenic disorders in other organs. This can incentivize companies to invest in therapies that can target indications that have better defined genotype-phenotype relationships, such as oncology or monogenic disorders in the retina or liver.

The potential of off-target effects also plays an important role in the risk-aversion to the investment in CNS *in vivo* genome editing therapeutics. A permanent off-target change to the DNA could lead to material consequences for the patient. It is possible that biotechnology companies are waiting for increased specificity of CRISPR and other tools before targeting the CNS. In fact, seven out of nine disclosed *in vivo* genome editing therapies treating CNS indications (Table 1) are using tools acting on transcriptional regulation which leads to transient changes in neuronal gene expression, rather than genome modifications.

In summary, overcoming some technical limitations that are specific for CNS, such as temporal and spatial control of tool expression, delivery and targetability (Wang et al., 2020); as well as accuracy and efficacy (Zhang et al., 2015) could increase the interest of biotechnology companies toward *in vivo* genome editing for CNS disorders, and therefore also increase investments and number of therapies in the clinic.

### Partnerships

Biopharmaceutical companies developing *in vivo* genome editing therapies and advanced therapeutics are partnering with other biotechnology companies in order to make progress on some of those key limitations. For example, the partnership with AskBio will enable Editas Medicine to leverage its knowledge and IP on capsid development and its AAV delivery system in order to overcome the aforementioned bottlenecks of *in vivo* genome editing in the CNS^5^. In the transient gene therapy space, Roche and Spark Therapeutics partnered with Dyno Therapeutics in order to use Dyno Therapeutics’ CapsidMap^TM^ platform to develop optimized AAV vectors for gene therapies targeting CNS and liver^6^. Those novel AAVs will have optimized tissue targeting and “immune-evading” properties.

Some CNS indications, however, have already an attractive commercial proposition. In fact, there are indications such as Huntington’s and ALS, for which there is a large therapeutic unmet need and the etiology is clear and are therefore suitable indications to be treated with *in vivo* genome editing. For this reason, large biopharmaceutical companies have partnered with genome editing companies to treat CNS disorders (Table 2).

**TABLE 2:** Licensing deals from co-developed *in vivo* genome editing and regulation CNS assets (excluding AskBio/Editas) https://investor.sangamo.com/news-releases/news-release-details/sangamo-announces-global-collaboration-novartis-develop-genomic.

Licensee	Licensor	Phase	Indication	Upfront ($m)	Milestone Payments (Up to $m)	Year
Pfizer	Sangamo Therapeutics	Pre-clinical	ALS/FTLD	12	150	2018
Takeda Pharmaceutical Company	Sangamo Therapeutics	Pre-clinical	Huntington’s	Unknown	Unknown	2019
Biogen	Sangamo Therapeutics	Pre-clinical	Tauopathies, Synucleinopathies (Inc., Parkinson’s disease), a neuromuscular target and up to nine other undisclosed neurological indication	350	2,370	2020
Novartis	Sangamo Therapeutics	Pre-clinical	Neurodevelopmental Disorders (Inc., Autism Spectrum Disorder)	75	720	2020

Sangamo has positioned itself as the leader in *in vivo* genome editing for CNS disorders with its ZFP-TF technology. With four large collaborations with Pfizer, Takeda Pharmaceutical Company (Takeda), Biogen and Novartis (Table 2), it has managed, at least publicly, to become the biopharmaceutical company with the largest amount of genome editing therapeutic assets for CNS indications.

All disclosed CNS *in vivo* genome editing therapeutics are in early stages, but their potential is reflected in the large partnering and licensing deals (Table 2).

Sangamo signed two collaboration agreements with Pfizer and Takeda for the development of therapies for ALS/FTLD and Huntington’s, respectively. Under the collaboration with Pfizer, Sangamo will receive a $12m upfront payment from Pfizer^7^. In this agreement, Sangamo will be responsible for developing ZFP-TF candidates and Pfizer responsible for research, development, manufacturing and commercialization for the ZFP-TF program. Sangamo is eligible to receive development and commercial milestones of up to $150m, as well as tiered royalties on net sales.

More recently, Sangamo announced a global collaboration with Biogen to develop gene regulation therapies for tauopathies including Alzheimer’s disease, for synucleinopathies including Parkinson’s disease, a third undisclosed neuromuscular disease target, and up to nine additional undisclosed neurological disease targets. Sangamo will use its ZFP-TF platform to develop these assets. Biogen paid $350m upfront with up to $2.37b in development, regulatory, and commercial milestone payments^8^. In July 2020, Sangamo and Novartis announced a global collaboration to develop and commercialize gene regulation therapies to address three neurodevelopmental diseases, including autism spectrum disorder. The target genes are undisclosed. Novartis will pay $75m to Sangamo as an upfront license fee payment with a potential $720m in other development and commercial milestone payments. The agreement also stipulates that Sangamo is eligible to receive a high single-digit to sub-teen double-digit royalties on net commercial sales arising from the collaboration^9^.

### Patenting and Licensing

The commercialization route for biologics and advanced therapeutics, including genome editing therapeutics, is different from that of small molecules. Small molecule developers usually do not require a license for a critical technology (such as genome editing tools) in order to commercialize a therapy. In the case of advanced therapeutics, such as the use of CRISPR, any academic or commercial institution would require a license to key IP in order to have “freedom-to-operate” and to commercialize its CRISPR-based therapeutic. This is a major barrier to entry since developing a *de novo* genome editing tool in order to avoid expensive CRISPR licenses requires years of fundamental research (Brinegar et al., 2017).

**FIGURE 1:**
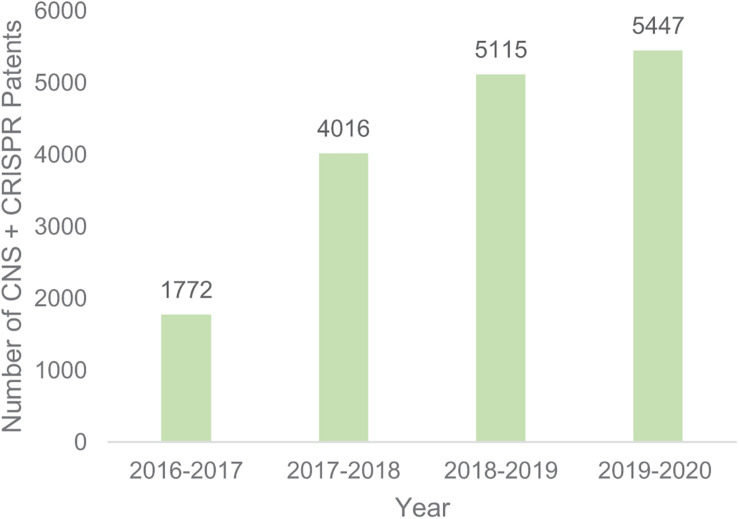
Number of patents filing that mention both CRISPR and CNS. Google patents search November 10 2020.

However, there are still 52,603 CRISPR patents filed globally (Google patents search 10/11/2020), of which 5,447 mention the CNS. The total number of patent filings that mention both CRISPR and CNS have been increasing since 2016 (Figure 1), demonstrating both the academic and institutional interest in the use of genome editing in the CNS.

For each CRISPR patent filed, there can be multiple licenses. For example, the Broad Institute licensed its key patents, non-exclusively, to The Monsanto Company (part of Bayer) for use in agriculture (StatNews, 2016^10^), but licensed it exclusively to Editas Medicine for human therapeutic use (Editas Medicine, 2014^11^).

### Conclusion and Future Perspectives

Although, as aforementioned, CRISPR/Cas9 tools can be designed and implemented much more easily than ZFPs, most of the preclinical studies that companies are running are based on ZFPs. This might be partially due to the more recent advent of CRISPR and the associated off-target effects, which have to be further tested. We now have several genome editing tools in our hands to really change the course of neurological disease treatment. Preclinical studies are promising and there are extensive efforts in the scientific community to find approaches to overcome the current barriers to developing a first in human genome editing therapeutic for CNS diseases. We envision that the next 5–10 years will be fundamental to understand whether we can completely eradicate some severe intractable neurological diseases using genome editing. The road to clinic is still full of hurdles but the speed of development in the field is one of the fastest ever seen in science.

## Author Contributions

PL, GC, and GL conceived the review and wrote the manuscript. All authors contributed to the article and approved the submitted version.

## Conflict of Interest

PL was employed by Hummingbird Ventures. The remaining authors declare that the research was conducted in the absence of any commercial or financial relationships that could be construed as a potential conflict of interest.

## Footnotes

1 www.clinicaltrials.gov 2020 Search 13 November 2020

2 https://media.bayer.com/baynews/baynews.nsf/id/Bayer-acquires-Asklepios-BioPharmaceutical-to-broaden-innovation-base-in-cell-and-gene-therapy

3 https://www.sangamo.com/pipeline

4 https://www.businesswire.com/news/home/20191031005255/en/Beam-Therapeutics-Announces-Collaboration-and-Exclusive-License-Agreement-with-Prime-Medicine-for-Prime-Editing-Technology

5 https://www.askbio.com/editas-medicine-and-askbio-enter-strategic-research-collaboration-to-explore-in~vivo-delivery-of-genome-editing-medicines-to-treat-neurological-diseases/

6 https://www.dynotx.com/news/press-releases/dyno-therapeutics-enters-collaboration-and-license-agreement-with-roche-to-develop-next-generation-aav-gene-therapy-vectors-for-cns-diseases-and-liver-directed-therapies/

7 https://www.pfizer.com/news/press-release/press-release-detail/sangamo_and_pfizer_announce_collaboration_for_development_of_zinc_finger_protein_gene_therapy_for_als

8 https://investors.biogen.com/news-releases/news-release-details/biogen-and-sangamo-announce-global-collaboration-develop-gene

9 https://investor.sangamo.com/news-releases/news-release-details/sangamo-announces-global-collaboration-novartis-develop-genomic

10 https://www.statnews.com/2016/09/22/monsanto-licenses-crispr/

11 https://ir.editasmedicine.com/news-releases/news-release-details/editas-medicine-licenses-genome-editing-technology-broad?ID=2125239&c=254265&p=irol-newsArticle
